# Botanical polysaccharides as veterinary vaccine adjuvants: structure, receptors, and translational limits

**DOI:** 10.3389/fimmu.2026.1982641

**Published:** 2026-10-12

**Authors:** Ran Guan, Chaoyun Yang

**Affiliations:** College of Animal Science, Xichang University, Xichang, China

**Keywords:** gut–immune axis, mucosal immunity, pattern recognition receptors, species-specific immunity, structure–activity relationship, traditional Chinese medicine polysaccharides, veterinary vaccine adjuvants

## Abstract

Intensive livestock production depends critically on vaccination, yet the licensed veterinary adjuvant repertoire remains narrow, being dominated by aluminium salts and mineral-oil emulsions that offer limited control over response quality and cause injection-site reactions and carcass losses. Polysaccharides from Traditional Chinese medicine (TCM), here referred to as TCM polysaccharides (TCMPs), have attracted attention as candidate adjuvants owing to their structural diversity, low toxicity, and mimicry of pathogen-associated molecular patterns. This review surveys immunomodulatory TCMPs by botanical origin and medicinal part, then reinterprets them structurally: adjuvanticity tracks glycosidic backbone type, molecular weight, branching, and substitution rather than taxonomic provenance. Mechanistically, adjuvant activity engages a multi-tiered cascade — pattern-recognition receptor activation (TLR2/TLR4, Dectin-1–Syk, NLRP3 inflammasome), dendritic-cell programming, modulation of the gut–immune axis and mucosal immunity, and engagement of T follicular helper-dependent humoral responses and, tentatively and as a hypothesis, tissue-resident memory T cells. Species-specific features are emphasised as potential, host-relevant mechanisms rather than established TCMP effects — including proposed Dectin-1-driven repolarisation of porcine alveolar macrophages, WC1+ γδ T cell activation in ruminants, and gut-associated lymphoid tissue in poultry — aspects poorly predicted by murine models. Critical appraisal nevertheless reveals substantial limitations: effects are most often reported for chemically undefined crude extracts, endotoxin contamination is rarely excluded, dose–response relationships are frequently non-monotonic, comparisons with licensed adjuvants are scarce, and batch-to-batch variability compromises reproducibility; biosafety claims rest largely on short-term observations. Translation to the field will depend on standardisation of structurally defined preparations, validated structure–activity relationships, rational particulate and mucosal delivery, DIVA compatibility, and economically viable extraction. The evidence positions TCMPs as a mechanistically attractive but analytically immature adjuvant class, whose contribution to antibiotic-free animal production will be determined less by new bioactive extracts than by analytical and regulatory rigour.

## 1 Introduction

The intensification of livestock production over the past four decades has concentrated large and genetically uniform populations within confined housing systems, and in doing so has raised the transmission efficiency of infectious agents to levels that were unattainable under extensive husbandry (1). Economic loss and compromised welfare follow directly from this arrangement. A second pressure has been superimposed upon the first: antimicrobial growth promoters, once relied upon as an informal buffer against subclinical infection, have been withdrawn or severely restricted across the major producing regions. Since global antimicrobial consumption in food animals had been projected to rise by two-thirds by 2030 in the absence of such intervention (2), the withdrawal was unavoidable; its consequence, however, is that prophylactic immunisation must now carry a share of the disease-control burden that it was never designed to bear alone. Vaccination remains the most cost-effective means of preventing virulent infectious disease (3), but the margin by which it can absorb this additional load is finite, and much of that margin is determined by adjuvant performance.

An adjuvant is a component administered with, or shortly before, an antigen in order to augment the immune response, whether by increasing its magnitude and duration or by redirecting it towards a preferred effector phenotype (4, 5). For poorly immunogenic formulations, and for animals whose responsiveness is constrained by age, stress, or concurrent infection, this choice is critical (6). It is also the point at which veterinary vaccinology is least well served. Adjuvant development has proved unusually attrition-prone, and candidates with convincing immunostimulatory activity in preclinical models have repeatedly been abandoned at later stages on grounds of reactogenicity rather than of insufficient potency (7). The licensed repertoire has expanded correspondingly slowly since the 1920s. In human vaccinology, the adjuvants that are available may be grouped by mechanism into antigen delivery systems (aluminium hydroxide, the MF59^®^ oil-in-water emulsion, AS03, lipid nanoparticles), immunostimulants such as the cytosine–phosphate–guanine oligodeoxynucleotide 1018, and combined formulations including AS01, AS02, and AS04 (8). Veterinary practice draws on a narrower selection still, being dominated by aluminium salts and mineral-oil emulsions.

Neither of these dominant classes is well matched to the requirements of contemporary livestock vaccination. Aluminium salts bias the response towards antibody production and afford little purchase on the mucosal and cytotoxic arms that mediate protection against respiratory and enteric pathogens (9–11). Mineral-oil emulsions are more potent but achieve this at a recognised cost, injection-site inflammation and granuloma formation having been documented in poultry and swine, with residual lesions detectable at slaughter and consequent trimming or downgrading of carcasses (9–12). Neither limitation is newly discovered, and neither has been resolved by incremental reformulation. The persistence of both explains the sustained interest in adjuvant chemistries drawn from outside the mineral and petrochemical traditions.

Natural polysaccharides are macromolecules distributed ubiquitously among plant, fungal, algal, bacterial, and animal cells, and anti-inflammatory (13), antioxidant (14), antiviral (15), antitumour (16), and immunomodulatory (17) activities have all been ascribed to them. Their candidacy as adjuvants, however, does not rest on this pharmacological breadth alone, which would in itself constitute a weak argument. It rests on a defined molecular basis: β-1, 3/1, 6-glucans, mannans, and certain arabinogalactans are structurally homologous to microbial cell-wall constituents, and are consequently recognised by the C-type lectin receptor Dectin-1 and by Toll-like receptors as pathogen-associated molecular patterns (18). The innate sensing pathways thereby engaged are the same as those exploited by conventional immunostimulants, and their sustained engagement has additionally been linked to metabolic and epigenetic reprogramming of myeloid progenitors, the phenomenon designated trained immunity (19). Plant-derived polysaccharides have further been reported to display lower toxicity than their bacterial counterparts (20), and their abundance, biocompatibility, biodegradability and aqueous solubility (21, 22) are practical advantages in a sector in which unit cost per dose is unforgiving. Among this group, the polysaccharides of Chinese medicinal herbs occupy a distinctive position, since documented clinical use in both human and veterinary medicine provides a tolerability precedent that synthetic candidates cannot offer; the *Astragalus* polysaccharide injection listed in the Chinese Pharmacopoeia for infectious bursal disease is the clearest illustration (23).

Set against this, the evidential base remains considerably less mature than the volume of publication would suggest, and it is this discrepancy that the present review is intended to address. Three difficulties recur. The first is analytical. Adjuvant activity is most often attributed to crude extracts of undefined composition, in which protein, phenolic, and nucleic acid residues persist and in which bacterial lipopolysaccharide is rarely quantified or excluded; given that endotoxin contamination at nanogram concentrations is sufficient to activate monocytes and dendritic cells, and that such contamination has been demonstrated in botanical preparations of comparable provenance, attribution of observed immunostimulation to the polysaccharide fraction itself is frequently presumptive rather than established (24). The second is translational. Mechanistic understanding derives overwhelmingly from murine and *in vitro* systems, whereas the innate compartments that govern responsiveness in target species diverge appreciably from the murine template: porcine alveolar macrophages differ in receptor repertoire and polarisation behaviour, ruminants maintain a large circulating population of WC1^+^ γδ T cells with no close murine equivalent (25), and avian protective immunity is disproportionately dependent upon gut-associated lymphoid tissue and the caecal tonsils (26). Extrapolation across these boundaries is not a formality. The third concerns dose and context. Reported dose–response relationships are frequently non-monotonic, and the same preparation has been shown to amplify T cell responses to one antigen while attenuating them to another, an observation that is difficult to reconcile with the notion of a generic immunostimulant. Direct comparison against licensed commercial adjuvants, which alone would establish practical relevance, is correspondingly rare. Earlier reviews in this field have, on the whole, enumerated bioactive extracts without engaging with these constraints.

The organisation of what follows reflects these considerations. Immunomodulatory TCMPs are first surveyed by botanical origin and medicinal part, in accordance with pharmacognostic convention, and the survey is then reinterpreted structurally, on the grounds that adjuvanticity tracks backbone linkage, molecular weight, branching, and substitution pattern rather than taxonomic provenance (27). Mechanism is examined next, proceeding from pattern recognition receptor engagement through dendritic cell programming and modulation of the gut–immune axis to the establishment of germinal centre-dependent humoral memory and tissue-resident memory T cell populations; throughout this account, findings obtained in target livestock species are distinguished explicitly from those inferred from rodent models. Species-specific application in swine, ruminants, and poultry is then considered, with particular attention to mucosal administration routes, for which the anatomical and immunological constraints differ markedly between monogastric and ruminant hosts (28). The review closes with an appraisal of the obstacles that continue to separate laboratory observation from field use, among them the standardisation of structurally defined preparations, batch-to-batch reproducibility, delivery system design, compatibility with strategies for differentiating infected from vaccinated animals (29), and the economic viability of extraction at industrial scale.

Literature search and study selection. References were identified in PubMed, Web of Science, and the China National Knowledge Infrastructure up to June 2026, using combinations of the terms ‘polysaccharide’, ‘Chinese medicine’, ‘vaccine adjuvant’, ‘veterinary’, ‘Dectin-1’, and ‘immunomodulation’. In the present review, the term ‘botanical’ is used inclusively to denote plant- and fungus-derived Traditional Chinese medicine materials; polysaccharides of animal or bacterial origin are outside its scope. Retrieved studies were classified as vaccine studies (defined antigen with immunological or protective endpoints), general immunomodulatory studies (no vaccine context), or non-veterinary (predominantly murine or *in vitro*) studies, and were graded according to the framework in **Box 1**. Studies reporting negative, null, or inconsistent findings were retained. Comparative terms such as ‘most’ or ‘the majority’ are used only where the retrieved literature supports them.

Box 1 **Evidence-grading framework used in this review.**

Tier 1 — target animal species, defined antigen, protective efficacy assessed (challenge or field endpoint). Tier 2 — target animal species, antigen-specific immune response assessed. Tier 3 — mouse vaccine model. Tier 4 — immunomodulatory, nutritional, or *in vitro* study without a vaccine context. Antibody titres, total IgA, and cytokine concentrations are reported separately from protective efficacy, pathogen burden, and clinical benefit; only Tier 1 evidence supports claims of protection.

## 2 Polysaccharides as vaccine adjuvants derived from Chinese medicine herbs and medicinal fungi

Immunomodulatory TCM polysaccharides (TCMPs) are structurally heterogeneous, and the literature has conventionally been organised according to biological origin and medicinal part, yielding four groups: root and rhizome, leaf and whole herb, fruit and seed, and fungal sources (**Figure 1**). This arrangement is adopted here for descriptive convenience, and because it corresponds to the pharmacognostic framework within which most primary studies were designed. It should not, however, be mistaken for a mechanistic classification. Medicinal part determines glycan composition only indirectly, through the metabolic role of the tissue concerned — storage organs accumulating fructans and starch-like glucans, photosynthetic tissues accumulating pectic material, fungal cell walls yielding β-1, 3/1, 6-glucans — and considerable structural overlap exists between groups. The structural determinants that actually govern adjuvanticity are therefore treated separately in Section 2.5.

**Figure 1:**
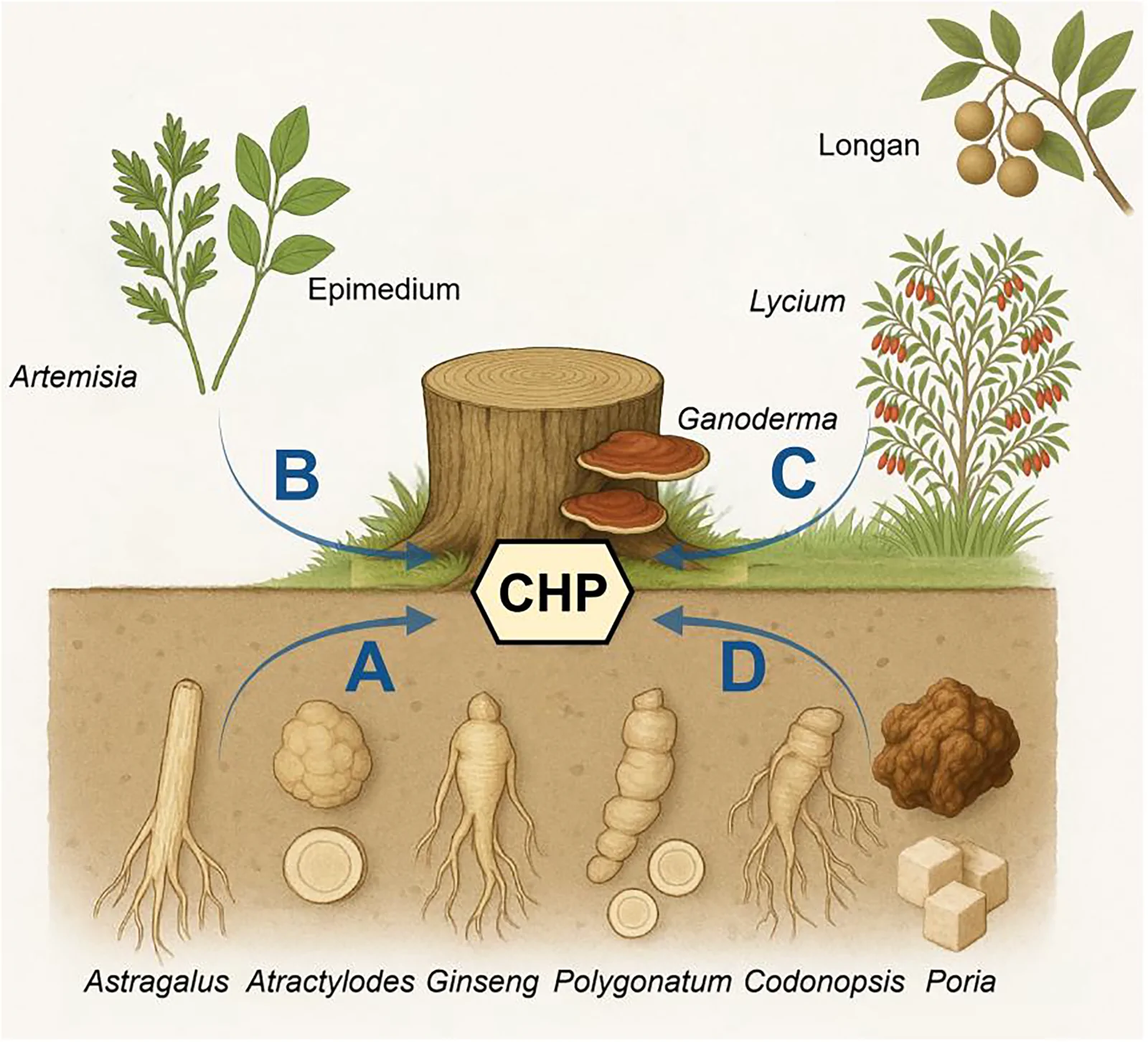
Classification of TCMPs as veterinary vaccine adjuvants by botanical origins and medicinal parts. The illustration is divided into two main sections: Lower Section: This section depicts subterranean parts, categorized into root and rhizome **(A)** and fungal **(D)** polysaccharides. Ganoderma, although classified under fungi, is uniquely represented as it grows on a tree stump. Upper Section: This section highlights aerial parts, with **(B)** representing leaves and whole herbs on the left, and **(C)** indicating fruits and seeds on the right. Created by BioRender.

### 2.1 Root and rhizome polysaccharides

Roots and rhizomes constitute the most intensively investigated category, comprising herbs traditionally classified as “Qi-tonifying” and “Blood-tonifying”. As perennating storage organs, these tissues accumulate polysaccharides in bulk, which accounts both for the high extraction yields reported and for the compositional profile typical of the group: neutral glucans and fructans of storage origin, accompanied by pectic arabinogalactans derived from the cell wall. The pharmacological attention devoted to this category should nevertheless be recognised as partly historical, reflecting the prominence of these herbs in classical formularies rather than any demonstrated superiority over aerial or fungal sources.

#### 2.1.1 Astragalus polysaccharides

*Astragali Radix* (AR, “Huangqi”), a medicinal and edible herb long employed to strengthen the constitution and promote convalescence (30), yields water-soluble polysaccharides (APS) with reported molecular weights spanning 10–1000 kDa (31). This range is itself informative: it reflects not a single macromolecule but a compositionally variable mixture whose principal constituents include a neutral α-1, 4/1, 6-glucan and an acidic arabinogalactan-rich pectic fraction, present in proportions that vary with cultivar, growing region, plant age, and extraction conditions (31). Antioxidantantitumour, ur and immunomodulatory activities have all been ascribed to APS (32–34). Its regulatory standing is unusual among candidate adjuvants, the APS Injection (“Huangqi Duotang zhusheye”) being listed in the Chinese Pharmacopoeia (2020 edition) for the treatment of infectious bursal disease, where enhancement of host immunity and antibody production is claimed.

Adjuvant efficacy has been documented across an exceptionally broad range of antigens, including vaccines against Newcastle disease virus (NDV) (35–38), foot-and-mouth disease virus (FMDV) (39, 40), hepatitis B virus (HBV) (41, 42), and infectious bronchitis virus (IBV). In murine models, sequential administration with HBV subunit and DNA vaccines elevated antigen-specific responses, an effect attributed to a reduction in regulatory T cell frequency. In chickens immunised against avian influenza virus (AIV), APS enhanced lymphocyte proliferation, raised antigen-specific serum IgG titres, modified circulating IL-2, IL-10, γ, FN-γ and TNF-α concentrations, and reduced viral replication (43, 44); improved survival following bacterial challenge has additionally been reported in fish (45, 46). The breadth of this list is frequently cited as evidence of versatility, but it warrants a more cautious reading, since a preparation reported to enhance responses to almost every antigen tested is as consistent with a non-specific inflammatory effect — or with residual endotoxin — as with defined adjuvanticity, and few of these studies characterised the fraction administered or reported endotoxin content.

Against this, one observation stands out as genuinely mechanistically informative. When APS was evaluated with inactivated influenza and recombinant SARS-CoV-2 vaccines, antigen-specific antibody titres were raised in both cases, yet T cell proliferation and Th-associated cytokine secretion were enhanced in the former and suppressed in the latter (47). Such bidirectional behaviour indicates that the outcome is determined jointly by the polysaccharide and by the antigenic context, and is difficult to reconcile with the notion of a generic immunostimulant. It also carries a practical implication that has been largely overlooked: adjuvant performance established for one vaccine platform cannot be assumed to transfer to another, and platform-specific validation is required.

#### 2.1.2 Ginseng polysaccharides

*Panax ginseng* (GP, “Renshen”), designated the “king of herbs”, has been employed for more than two millennia to tonify Qi and restore physiological equilibrium (48). The polysaccharides of the root (GPS) are heterogeneous, comprising starch-like glucans together with pectin-like acidic polysaccharides (49); the immunological activity is generally attributed to the acidic (pectic) fraction rather than to the neutral starch-like glucans, a distinction that is seldom preserved when crude GPS preparations are evaluated. Unlike the ginsenosides, GPS exhibits negligible haemolytic toxicity, and antioxidant, anti-ageing and immunomodulatory effects have been reported. Improved vaccination outcomes have been described against FMDV (50), AIV (43), porcine reproductive and respiratory syndrome virus (PRRSV) (51), with elevated antigen-specific serum IgG titres, enhancedproliferation, roliferation and coordinate upregulation of Th1-type (IFN-γ, IL-2) and Th2-type (IL-4, IL-10) transcripts (52). Simultaneous elevation of both cytokine sets is commonly interpreted as balanced enhancement of adaptive immunity; it may equally reflect generalised lymphocyte activation, and in the absence of protectdata, challenge data the two interpretations cannot be distinguished. The comparative absence of haemolytic activity nevertheless represents a tangible formulation advantage over saponin-based adjuvants such as QS-21, for which haemolysis and local reactogenicity have constrained veterinary application (53).

#### 2.1.3 Codonopsis pilosula polysaccharides

Codonopsis pilosula (CP, “Dangshen”) is widely cultivated and frequently substituted for ginseng on economic grounds, being valued as a Qi-tonifying herb held to strengthen splenic function and nourish the lungs (54). Its polysaccharides (CPP), composed principally of galacturonic acidglucose, se and arabinose, have attracted interest for prebiotic-like and immunomodulatory properties (55). In a bovine viral diarrhoea virus (BVDV) model, CPP inhibited viral adsorption to bovine macrophages and counteracted virus-induced immunosuppression, phagocytic activity being enhanced and expression of CDCD80, CD80 and CD86 upregulated (56); virus-associated cellular damage was subsequently reported to be attenuated through inhibition of apoptosis, tentatively ascribed to modulation of Bcl-xL and caspase-3 signalling (56). Dietary supplementation of growing–finishing pigs with a CPP–APS mixture increased average daily gain and elevated antibody titres against type O FMDV relative to unsupplemented controls (57). It should be noted that the last of these observations concerns oral supplementation of a two-component mixture in the context of general growth performance; attribution of the antibody effect specifically to CPP, or indeed to adjuvant action rather than to improved nutritional status, is not supported by the design employed. The high galacturonic acid content of CPP nonetheless aligns it with the pectic polysaccharides discussed in Section 2.5, for which complement activation and Peyer’s patch targeting have been more firmly established.

#### 2.1.4 Rhizoma Atractylodis Macrocephalae polysaccharides

*Rhizoma Atractylodis Macrocephalae* (RAM, “Baizhu”), the rhizome of *Atractylodes macrocephala* Koidz.yields polysaccharides (RAMP) composed of rhamnose, arabinose, xylose, mannose, glucose, and galactose (58, 59). Immunomodulatory, antitumour, antioxidant, hepatoprotective, and hypoglycaemic activities have been reported (60, 61), and adjuvant effects have been demonstrated with FMDV and NDV vaccines and with ovalbumin (OVA) as a model antigen (62–64).

RAMP is distinguished within this group by documented efficacy following oral administration, a route of considerable practical importance in intensive production, where individual injection is labour-intensive and stressful. Oral administration in mice increased serum FMDV-specific IgG and its IgG1, IgG2a, IgG2b, and IgG3 subclasses, together with total intestinal IgA, and promoted antigen-recalled splenocyte proliferation, indicating engagement of the gut-associated lymphoid tissue rather than simple systemic exposure. Parenteral co-administration with ND vaccine elevated NDV-specific serum IgG, stimulated peripheral blood T lymphocyte proliferation, increased the CD4^+^/CD8^+^ ratio, promoted progression of lymphocytes into S and G2/M phases, and attenuated immune organ atrophy induced by immunosuppressive viruses (65). Delivery engineering has been applied to this preparation: encapsulation into a nanostructured lipid carrier (PAMK-NLC) outperformed the free polysaccharide with OVA in mice, dendritic cell maturation, antigen-specific splenocytproliferation, on and T lymphocyte activation being more strongly enhanced, alongside higher serum OVA-specific IgG and TNF-α, IFγ, -γ and IL-12 concentrations (66). This comparison is instructive in a field where formulation variables are rarely isolated, since it establishes that particulate presentation contributes measurably beyond the intrinsic activity of the glycan itself (PAMK-NLC is the specific nanostructured-lipid-carrier formulation of Atractylodes polysaccharide, referred to elsewhere in this review as RAMP; the two names denote the same starting material.).

A methodological caveat attaches to the reported molecular weight of RAMP, variously given as 1000–4000 Da (58, 59). Oligosaccharides of this size lie well below the multivalent threshold generally required for cross-linking of pattern recognition receptors, and the figure is difficult to reconcile with the receptor-mediated mechanisms proposed elsewhere for this preparation; the discrepancy most probably reflects differences in calibration standards or in the fraction analysed, and independent characterisation is required.

#### 2.1.5 Polygonatum sibiricum polysaccharides

*Polygonatum sibiricum* (PS, “Huangjing”), a classical “Yin-tonifying” herb, yields highly heterogeneous polysaccharides (PSP) whose activities depend markedly on species and on processing. A low-molecular-weight fraction (PKP2-1, approximately 7.4 kDa) isolated from *Polygonatum kingianum* behaved as a targeted bifidogenic agent rather than as a conventional immunostimulant, increasing the relative abundance of *Bifidobacterium* from 5.38% to 42.66% during *in vitro* faecal fermentation (67). Whether such a prebiotic effect translates into mucosal adjuvanticity *in vivo* remains untested, and the inference should be regarded as a hypothesis rather than a demonstrated property.

Of greater immediate relevance is the effect of traditional processing. Comparison of raw and “nine steaming–nine sun-drying” processed *Polygonati Rhizoma* revealed a structural transformation from β-fructans to β-galactans, the processed polysaccharide (PPR-N2) displaying superior anti-senescence activity in H_2_O_2_-treated fibroblasts, with restoration of mitochondrial membrane potential, alleviation of G1-phase arrest and suppression of senescence-associated secretory phenotype factors (IL-6, IL-8, CXCL-10) (68). Beyond its pharmacological interest, this finding is methodologically significant for the field as a whole: it demonstrates that a defined thermal treatment alters glycan backbone chemistry and, with it, biological activity, thereby identifying processing history as a variable that must be specified if preparations are to be compared meaningfully between laboratories. Its relevance to vaccination is prospective — an application in stressed or aged animals, in which immunosenescence limits vaccine responsiveness, has been proposed but not yet examined experimentally.

### 2.2 Polysaccharides from leaves and whole herbs

Polysaccharides obtained from leaves and whole herbs have received considerably less attention as adjuvant candidates than those of roots and rhizomes, and the imbalance is difficult to justify on biochemical grounds. Aerial tissues serve photosynthetic and structural rather than storage functions, and their polysaccharide complement differs accordingly: whereas underground organs accumulate starch-like glucans and fructans, leaves and herbaceous stems are enriched in cell-wall pectic material — homogalacturonan, rhamnogalacturonan, and arabinogalactan-proteins — together with hemicellulosic xyloglucans (69). Since galacturonic acid content and rhamnogalacturonan-II domains are among the structural features most consistently associated with complement activation and macrophage stimulation, aerial tissues are, if anything, the more promising source. The herbs in question are traditionally classified as “heat-clearing”, “detoxifying” or “Yang-tonifying”, categories that map loosely onto anti-inflammatory and cell-mediated immune effects; such correspondences are, however, interpretive and should not be treated as mechanistic predictions.

#### 2.2.1 Epimedium polysaccharides

*Epimedium* spp. (ES, “Yinyanghuo”), a principal “kidney-Yang” tonifying herb, yields polysaccharides (EPS) that have been characterised chiefly as modulators of the gut–immune axis rather than as direct immunostimulants (70, 71). Two lines of action have been described. Intestinal barrier integrity was restored through activation of the Keap1/Nrf2 antioxidant pathway and induction of autophagy via AMPK/mTOR signalling in a murine colitis model (70), while microbial composition was reshaped by enrichment of *Akkermansi* and concurrent suppression of Th17 differentiation in a model of *Porphyromonas gingivalis*-exacerbated intestinal inflammation (71). Both effects are relevant to mucosal immune competence, though it should be noted that neither study involved vaccination, and the extension from barrier repair to adjuvanticity remains an inference rather than an observation.

A more distinctive property is the capacity of an *Epimedium brevicornu* glucan to self-assemble and spontaneously encapsulate hydrophobic flavonoids, including icariin, with consequent improvement in their bioavailability (72). This is of interest less as a therapeutic finding than as a formulation principle, since it identifies a polysaccharide that may function simultaneously as immunostimulant and as delivery vehicle — a dual role that would otherwise require conjugation to a separate carrier. Whether antigen can be accommodated in the same manner as small hydrophobic ligands has not been examined, and the steric requirements differ substantially.

Direct veterinary evidence is limited to a single formulation. Administration of sulfated EPS together with APS to newborn piglets upregulated NF-κB and IRF-3 expression, accompanied by enhanced T lymphocyte proliferation (73). Because the two polysaccharides were given in combination, the contribution of EPS cannot be resolved, and the sulfated derivative rather than the native glycan was employed; sulfation is known to alter charge density, conformation and receptor affinity, so that the result cannot be transferred to unmodified EPS. The observation is nonetheless notable in demonstrating activity in neonates, a population in which conventional adjuvants perform poorly owing to immunological immaturity and maternal antibody interference.

#### 2.2.2 Artemisia annua polysaccharides

*Artemisia annua* L. (AA, “Qinghao”), a classical “heat-clearing” herb, yields polysaccharides (AAPs) for which a coordinated immune and antioxidant action has been proposed. In lambs receiving dietary supplementation, intestinal immunoglobulin secretion (sIgA, IgG) was increased and the cytokine profile shifted towards IL-4, with IL-2 and IL-1β concentrations maintained, the response being attributed to the TLR4–MyD88–NF-κB cascade and maximal at an inclusion rate of 1000 mg/kg (74). It must be noted that this study administered a whole aqueous extract rather than an isolated polysaccharide, so the effects cannot be attributed to the polysaccharide fraction alone; the fraction-resolved data below are the appropriate basis for structure–activity inference. Antioxidant indices moved in parallel, Nrf2 and HO-1 expression being elevated with concomitant Keap1 inhibition, GSH-Px and CAT activities increased, and malondialdehyde reduced, while DPPH and ABTS^+^ scavenging rates of 68–73% were recorded *in vitro* (74, 75). The existence of a dietary optimum is worth emphasising, since it indicates a non-monotonic dose–response and undermines any assumption that higher inclusion confers greater benefit; this is immunomodulatory rather than vaccine-based evidence (Tier 4).

AAPs are additionally distinguished by the clearest structure–activity relationship reported among the preparations reviewed here. Across three homogeneous acidic fractions, galacturonic acid content ranged from 8% to 28%, and anticomplement activity tracked this parameter directly: the high-uronic fraction AAP01-2 (28% GalA) inhibited haemolysis with a CH_50_ of 0.360 mg/mL through interaction with C1q, C3, C4, C5, and C9, whereas low-uronic fractions were inactive (76). Separately, a high-molecular-weight fraction (1684 kDa) enriched in arabinose and galactose with β-glycosidic linkages induced a sixteen-fold increase in IL-6 secretion from macrophages with minimal cytotoxicity (75). Because activity was compared between purified fractions of defined composition rather than between crude extracts, these data satisfy an evidential standard rarely met elsewhere in this literature, and they support the argument developed in Section 2.5 that uronic acid content and linkage pattern, rather than plant origin, determine adjuvant potential. The qualification is that anticomplement activity and macrophage activation are *in vitro* surrogates; neither has been evaluated with a vaccine antigen in a target species, and it should further be recognised that inhibition of complement is not straightforwardly an immunostimulatory property, complement being required for the follicular retention of antigen and for optimal B cell activation.

#### 2.2.3 Other leaf-derived polysaccharides

Beyond these two examples, several leaf polysaccharides have been characterised immunologically but have not yet been assessed as adjuvants; this group remains comparatively under-studied within the field. A polysaccharide isolated from the leaves of Isatis indigotica, a herb of long standing in poultry practice, displayed immunomodulatory activity that increased markedly after controlled acid degradation, the native high-molecular-mass form being comparatively inactive (77) — an observation consistent with the molecular mass dependence discussed in Section 2.5. Dietary Eucommia ulmoides polysaccharide raised circulating lymphocyte counts and serum IgA, IgG2a and IFN-γ concentrations in pigs (78), although the preparation employed was of low purity and was administered at percentage inclusion rates, so the evidence pertains to feed supplementation rather than to adjuvant action in the strict sense. Polysaccharide MOP-3 from Moringa oleifera leaves has been structurally defined and shown to enhance macrophage and lymphocyte responses *in vitro* (79), and pectin-rich fractions of Taraxacum species stimulate cytokine secretion in murine macrophages (80). In each case the immunological groundwork exists while adjuvant evaluation with a defined antigen, in a target species, does not; given that leaf biomass is harvestable without destroying the plant and is renewable annually (Section 5.6), this gap appears to reflect research habit rather than any intrinsic limitation of the material.

### 2.3 Polysaccharides from fruits and seeds

Fruits and seeds occupy a particular position in this survey, not because their polysaccharides are chemically privileged, but because most of the source materials are formally recognised as food, and their long dietary record lowers the regulatory threshold for use in feed-integrated or orally delivered formulations. Chemically, storage and reproductive organs differ from roots in a predictable way: the cell walls retain a high proportion of pectic material, so acidic heteropolysaccharides rich in galacturonic acid, arabinose, and galactose typically dominate, whereas neutral storage glucans are a minor fraction (81). Because galacturonan and rhamnogalacturonan backbones are poorly digested by mammalian and avian glycosidases yet readily fermented in the hindgut, these preparations tend to act on two fronts at once — direct engagement of mucosal antigen-presenting cells and indirect modulation through microbial metabolites (82). That duality is attractive but analytically inconvenient, since an oral study cannot easily distinguish a receptor-mediated adjuvant effect from a microbiota-mediated one unless germ-free or antibiotic-depleted controls are included, and such controls remain rare in the veterinary literature.

#### 2.3.1 Lycium barbarum polysaccharides

*Lycii Fructus* (LF, “Gouqizi”), the dried ripe fruit of *Lycium barbarum* L. (Solanaceae), is used in Chinese medicine to nourish the liver and improve vision, and is among the best-characterised medicine–food materials (83). The polysaccharide fraction (LBP) is not a single molecule but a family of glycoconjugates: arabinogalactan-protein and rhamnogalacturonan I domains predominate, with reported molecular masses spanning roughly 10–200 kDa depending on extraction and fractionation, and a peptide moiety that contributes materially to immunological activity (84, 85). Failure to report which fraction was tested is the principal reason why LBP dose–response data from different laboratories cannot be pooled.

Adjuvant activity has nonetheless been demonstrated with reasonable consistency. Co-administration of LBP with a *Chlamydophila abortus* DNA vaccine raised specific antibody titres, IL-2, IFN-γ, and TNF-α production, and splenic T-cell proliferation in mice, and accelerated chlamydial clearance. With a recombinant protein antigen, LBP enhanced T-cell-dependent antibody responses in association with expanded follicular helper T-cell populations (86), which is mechanistically informative because Tfh expansion implies action on germinal-centre dynamics rather than simple depot formation. Dietary LBP improved growth performance and several immune indices in broilers (87), although antibody responses to vaccination were not the primary endpoint in that work and the effect size was modest.

The more instructive line of evidence concerns delivery. Free LBP administered parenterally is cleared rapidly and distributes poorly to draining lymph nodes; encapsulation in simple nanoliposomes increased both humoral and cell-mediated responses in mice and conferred adjuvant activity against porcine circovirus type 2 antigen (88, 89), and the mechanism was subsequently traced to enhanced murine dendritic-cell maturation with up-regulated co-stimulatory molecule expression (90). Notch signalling has also been implicated in LBP-driven dendritic-cell maturation in a tumour context (91). Two reservations apply. First, all of the work summarised here, including the PCV2-adjuvanticity experiment, was performed in mice; the PCV2 antigen is of porcine origin but the experimental host was murine, so this is a Tier 3 observation, and the mechanistic account remains inferential for pigs, in which alveolar and monocyte-derived dendritic cells differ from their murine counterparts in receptor repertoire and cytokine output. Second, the liposomal formulations that perform best are also those in which the carrier itself is immunologically active, and few studies include an empty-liposome arm of matched lipid composition, leaving the contribution of the polysaccharide per se imprecisely bounded.

#### 2.3.2 Schisandra chinensis polysaccharides

The fruit of *Schisandra chinensis* (Turcz.) Baill. (“Wuweizi”) yields acidic polysaccharides (SCP) in which galacturonic acid and arabinose are prominent (92). SCP preparations increase macrophage phagocytosis and nitric oxide release *in vitro* via TLR4-mediated activation (93); these are *in vitro* immunomodulatory observations without a vaccine context (Tier 4). The magnitude of these effects is generally smaller than that reported for Astragalus polysaccharides under comparable regimens, and the SCP literature is further complicated by the fact that lignan-rich extracts of the same fruit possess their own immunomodulatory and hepatoprotective actions; unless lignan removal is documented, attribution to the carbohydrate fraction is insecure. The one poultry study sometimes grouped with these materials concerns a different preparation (Glycyrrhiza polysaccharides, which improved and prolonged the response of chickens to Newcastle disease vaccination (94)) and is discussed under poultry (Section 4.3) rather than as evidence for SCP.

#### 2.3.3 Seed-derived polysaccharides

Seeds have received less attention than fleshy fruits, and the available evidence is fragmentary rather than absent. Polysaccharides from Cuscuta chinensis Lam. seeds exhibit immunostimulating and antioxidant activity *in vitro* (95), and Cuscuta chinensis preparations ameliorate cyclophosphamide-induced immunosuppression (96). Polysaccharides recovered from fruit-processing by-products are increasingly recognised as low-cost sources of immunomodulatory material (97). Such by-product streams matter for the economics discussed in Section 5, since raw-material cost is a decisive constraint on veterinary adjuvant deployment and seed cake is available at a small fraction of the price of cultivated root. What is presently lacking is any seed-derived preparation tested as a vaccine adjuvant in a target livestock species against a licensed comparator; until that gap is filled, the group should be regarded as a promising but unvalidated reservoir.

### 2.4 Fungal polysaccharides

Fungal polysaccharides differ from the plant materials above in a way that has genuine mechanistic consequence. Their dominant structural motif — a β-(1→3)-linked glucan backbone bearing β-(1→6) branches — is absent from vertebrate tissue and is therefore treated by the innate immune system as a microbe-associated molecular pattern, recognised principally by Dectin-1 (CLEC7A) and complement receptor 3, with contributions from scavenger receptors, lactosylceramide and, for some preparations, TLR2 (98). This defined receptor axis makes fungal glucans the most tractable subclass for structure–activity analysis and explains why their potency correlates with parameters that can be measured: chain length, degree branching, ing and the capacity to adopt a triple-helical conformation.

One qualification deserves emphasis at the outset, because it is routinely overlooked in the veterinary literature. The chicken genome lacks a clear CLEC7A (Dectin-1) orthologue, so the Dectin-1/Syk pathway that dominates β-glucan sensing in mammals cannot be assumed to operate in poultry (99); the receptor(s) responsible for avian β-glucan recognition are not established (Section 3.4). Mechanistic conclusions drawn from murine or porcine Dectin-1 signalling cannot therefore be transferred to poultry, even where the phenotypic outcome — improved antibody titre after vaccination — appears similar.

#### 2.4.1 Ganoderma lucidum and G. sinense polysaccharides

*Ganoderma*, listed in the Chinese Pharmacopoeia as “Lingzhi” and derived from *Ganoderma lucidum* or *G. sinense*, contains triterpenoids, nucleosides, and alkaloids alongside its polysaccharides (100). The carbohydrate fraction (GLP/GSP) is heterogeneous, comprising branched β-glucans together with glycoprotein-bearing heteropolysaccharides, and exerts immunomodulatory effects *in vitro* and *in vivo* (101, 102). Reported activities include lymphocyte proliferation, dendritic-cell maturation, enhanced macrophaphagocytosis, sis and increased cytokine secretion (103–105).

Adjuvant application has been demonstrated most clearly in poultry: oral GLP increased peripheral blood lymphocyte proliferation and serum antibody titres induced by Newcastle disease vaccine. Because free polysaccharides are rapidly cleared and poorly targeted, a liposomal formulation (GLP-Lip) was developed; in mice immunised with ovalbumin, it promoted dendritic-cell maturation and biased the response towards a Th1 profile, with elevated IFN-γ and IgG2a (106). This is a murine (Tier 3) observation, and the IgG2a result in particular is not avian evidence. The Th1 bias is the practically relevant finding, since it addresses the principal deficiency of aluminium salts in veterinary vaccines against intracellular pathogens. It should be noted, however, that triterpenoid carry-over is a real risk in Ganoderma preparations obtained by hot-water extraction followed by ethanol precipitation alone, and that G. lucidum and G. sinense are treated as interchangeable in much of the literature despite differing polysaccharide profiles.

#### 2.4.2 Lentinan and other mushroom β-glucans

Lentinan, the purified β-(1→3)-glucan with β-(1→6) branches from *Lentinula edodes*, is the most structurally defined polysaccharide in this review and the only one with a licensed clinical precedent in human medicine, which makes it a useful reference standard rather than merely another candidate (107). As a defined β-glucan, it has been examined mechanistically in pigs: β-glucan enhanced T-cell IFN-γ responses in cells derived from PRRSV-infected pigs *in vitro* (108) — a mechanistic observation without a vaccine context (Tier 4), and not a demonstration that lentinan combined with an antigen increases antibody titres. In chickens, mushroom and herb polysaccharides enhanced cellular and humoral immune responses (109). Comparable β-glucans from *Grifola frondosa* and *Pleurotus* spp. show the same qualitative pattern with lower reported potency, consistent with their lower degree of β-(1→6) branching.

Conformation is decisive for this group. Lentinan is a triple helix in water but dissociates into single chains in dimethyl sulphoxide or under alkaline conditions, and the single-chain form is substantially less active because Dectin-1 clustering — the trigger for Syk recruitment — requires a multivalent, rigid ligand (110). Studies that report activity without specifying solvent history or aggregation state are consequently difficult to interpret, and this single variable plausibly accounts for a large share of the discrepancies attributed elsewhere to species or dose.

#### 2.4.3 Cordyceps and Poria polysaccharides

Polysaccharides from cultivated *Cordyceps militaris* and *Ophiocordyceps sinensis* comprise mannoglucans and galactomannans rather than simple β-glucans, and engagement of the mannose receptor is therefore likely to complement Dectin-1 signalling (111). In chickens, C. militaris polysaccharides improved the immune response to Newcastle disease vaccination (112) (Tier 2); the earlier claim of activity against FMDV antigen was not supported by this source and has been removed. *Poria cocos* yields pachyman, a largely unbranched β-(1→3)-glucan that is water-insoluble and poorly active in native form; chemical derivatisation (phosphorylation, as characterised in the cited stud) improves solubility and confers macrophage-activating activity (113). (The modification previously described here as carboxymethylation/debranching was inconsistent with the cited source and has been corrected.) Pachyman is thus the clearest illustration in this field that immunological inactivity may reflect physical inaccessibility rather than an unsuitable primary structure, and that chemical modification is not cosmetic but constitutive of activity, while also illustrating that different derivatisations are distinct chemical entities requiring their own characterisation and safety data.

### 2.5 Structural determinants of adjuvant activity

The botanical arrangement adopted above is a narrative convenience, and its limitations become apparent once the preparations are compared across categories: *Poria* pachyman and lentinan share a backbone yet differ by orders of magnitude in activity, whereas pectic fractions from *Astragalus* root and *Lycium* fruit, taxonomically and anatomically unrelated, converge on similar rhamnogalacturonan architectures and similar receptor usage (114). Activity tracks structure, not organ of origin, and four parameters carry most of the explanatory weight.

Linkage type and receptor assignment. β-(1→3)/(1→6)-glucans signal predominantly through Dectin-1 and CR3; pectic rhamnogalacturonan I domains, particularly the arabinogalactan side chains, engage TLR4 in a manner distinguishable from lipopolysaccharide signalling by their MyD88 dependence profile and by insensitivity to polymyxin B (114); mannans and galactomannans recruit the mannose receptor and DC-SIGN homologues; α-(1→4)-glucans of storage type are, with few exceptions, inactive. The corollary is that “polysaccharide adjuvant” is not a pharmacological category, and that preparations dominated by different linkages should not be expected to be interchangeable in a formulation.

Molecular mass. Activity is characteristically non-monotonic in the systems examined. Fragments below roughly 5–10 kDa are generally too short to cross-link receptors, while material above several hundred kilodaltons aggregates, diffuses poorly from the injection site, and may be sequestered by resident macrophages before reaching the draining lymph node; intermediate masses are often optimal, though the window differs by receptor and by route (115). These thresholds are empirical rather than universal, and their applicability across different polysaccharide classes has not been established. In this connection, reports of potent receptor-mediated activity by preparations of 1–4 kDa — as claimed for certain *Atractylodes macrocephala* fractions (63) — are difficult to reconcile with the multivalent cross-linking that the proposed mechanisms require. Rather than assuming a calibration or unit error, this discrepancy should be treated as an open question: the original size-determination method, the size distribution, the aggregation state and the biological endpoint in question each require direct examination, and the value should not be propagated until this is done.

Branching, substitution, and charge. Within the glucan series, a degree of β-(1→6) substitution of approximately 0.2–0.4 is associated with maximal Dectin-1 clustering in the systems examined, with activity falling on either side (110); whether this optimum is general across glucans is not established. Among acidic polysaccharides, uronic acid content correlates with complement activation and with adhesion to the mucus layer, which is consistent with the anticomplementary and mucosal activities reported for *Artemisia annua* fractions (76) and for pectic Lycium domains. Deliberate derivatisation — sulphation, carboxymethylation, selenylation, phosphorylation — alters charge density and solubility and frequently increases potency, as in sulphated Epimedium polysaccharide (73) and phosphorylated pachyman (113); it also creates a new entity whose safety cannot be inferred from the dietary history of the parent material, a point with obvious regulatory implications.

Conformation and supramolecular state. Triple-helical and rod-like conformers are more active than random coils of identical composition, and self-assembled particulate forms combine receptor cross-linking with depot behaviour (110). Because conformation depends on extraction temperature, solvent, ionstrength, gth and storage, two batches with identical monosaccharide composition and average molecular mass may differ substantially in activity.

Progress in this area is limited less by conceptual difficulty than by reporting practice. Most veterinary studies characterise their material by monosaccharide composition and total sugar content alone, omitting linkage analysis, dispersity, conformational state, and — critically — endotoxin quantification, without which any TLR4-dependent effect observed with a plant extract is ambiguous (116). A minimum data set comprising molecular mass distribution, glycosidic linkage assignment, uronic acid and protein content, endotoxin concentration below a stated limit, and receptor dependence tested with blocking antibodies or knockout cells would allow the accumulated observations to be compared rather than merely counted, and is a prerequisite for the rational formulation discussed in Section 5.

## 3 Mechanisms underlying the adjuvant activity of Chinese medicine polysaccharides

Mechanistic accounts of polysaccharide adjuvanticity are frequently assembled retrospectively: an immunological outcome is observed, a receptor is nominated, and a signalling cascade is imported from the innate immunity literature. The resulting narratives are internally coherent yet often untested in the species and by the route in which the adjuvant effect was recorded. The framework set out below is therefore organised around four sequential events — recognition at the level of innate receptors, functional maturation of antigen-presenting cells, shaping of the adaptive response including its mucosal arm, and the physical fate of the material in tissue — and each is qualified by the strength of the evidence available for livestock rather than for laboratory rodents.

### 3.1 Recognition by pattern recognition receptors

Immune activation by Traditional Chinese medicine polysaccharides (TCMPs) begins with recognition by pattern recognition receptors (PRRs) on dendritic cells and macrophages. These molecules are commonly described as functional mimics of pathogen-associated molecular patterns, but the analogy requires care: unlike lipopolysaccharide or flagellin, which bind a single receptor complex with defined stoichiometry, most TCMP preparations are polydisperse, multivalent, and conformationally heterogeneous, and can consequently engage several receptor families in parallel (98, 117). Multipoint attachment can amplify the activation signal, but the inference that must not be drawn is the reverse one: receptor redundancy means that a single-gene knockout may not abolish all activity, yet this does not license the advance dismissal of negative blocking or knockout results. Where a response is not materially altered under conditions of adequate receptor blockade, this is evidence against a major role for that receptor under the tested conditions; the magnitude of any residual activity should be reported quantitatively and interpreted against blocking efficiency, receptor expression, cell type, and alternative pathways (**Figure 2**).

**Figure 2:**
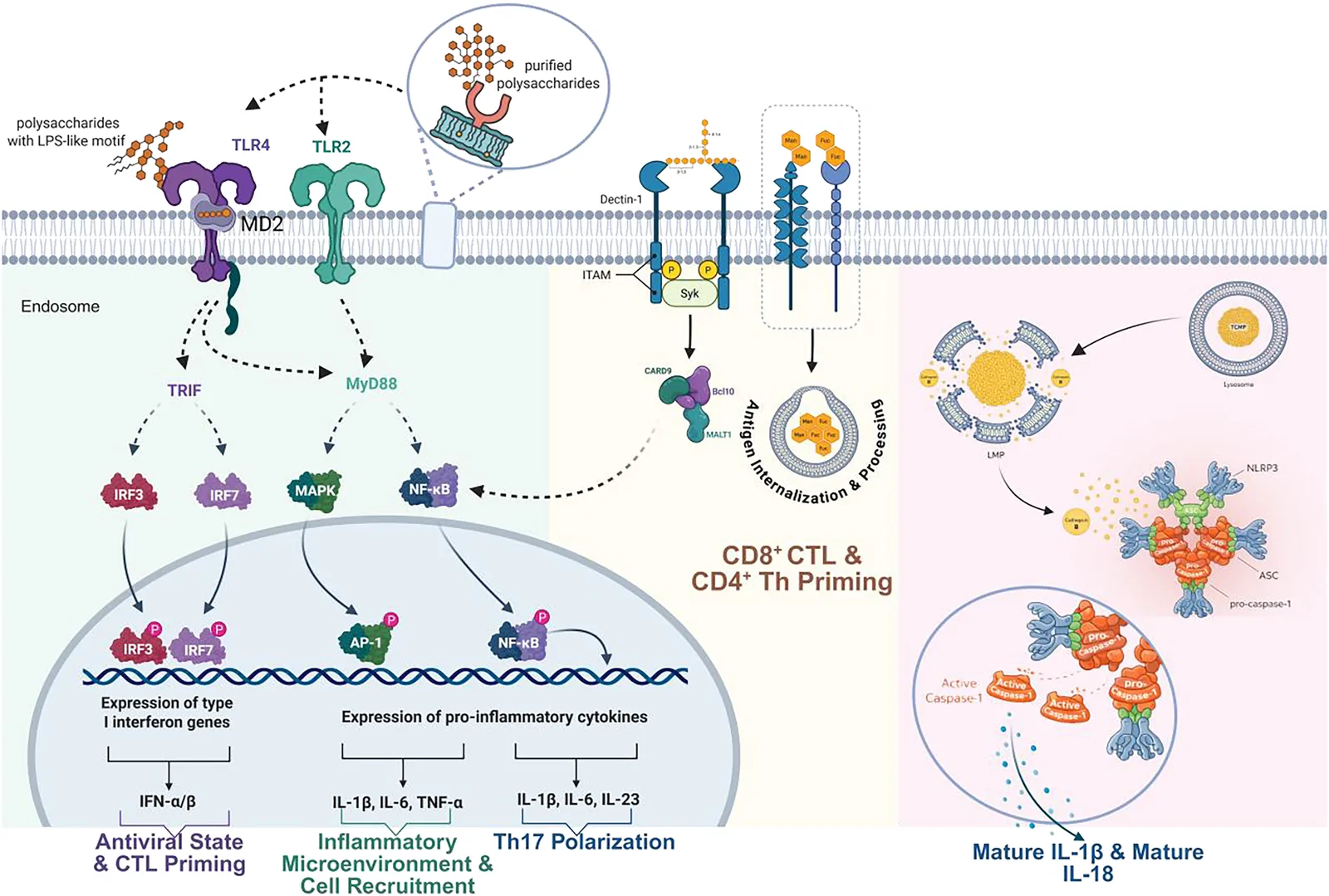
Mechanisms of immune activation by TCMPs through pattern recognition receptors (PRRs). This illustration depicts the mechanisms by which TCMPs activate various immune pathways through PRRs. The interaction of polysaccharides with TLR2 and TLR4 triggers downstream signaling, leading to the activation of antiviral responses and CTL priming through either the MyD88-dependent or TRIF-dependent pathways. This results in the expression of type I interferon genes, establishing an antiviral state. Additionally, Dectin-1, recognizing β-glucan-rich polysaccharides, activates a Syk-dependent signaling cascade, promoting the production of pro-inflammatory cytokines such as IL-1β, IL-6, and TNF-α, creating an inflammatory microenvironment conducive to Th17 polarization. The NLRP3 inflammasome is activated by aggregated/particulate polysaccharide material and associated danger signals — rather than by direct recognition of polysaccharide epitopes — triggering the maturation of IL-1β and IL-18, which enhances cellular immunity and adaptive responses. This mechanism highlights the synergistic activation of TCMPs through multiple receptors and signaling pathways, emphasizing their potential as effective vaccine adjuvants. Created by BioRender. Evidence code: solid arrows denote mechanisms with direct experimental support in the cited systems; dashed arrows denote inferred or general-immunology mechanisms not demonstrated for TCMPs. NLRP3 is depicted as activated by particulate material and associated danger signals, not as directly recognising polysaccharide epitopes; the polysaccharide→TLR2/TLR4 arrows should be read in the light of the endotoxin caveats in Section 3.1.1.

#### 3.1.1 Toll-like receptors

TLR4 is the receptor most often nominated for plant-derived TCMPs, including Astragalus polysaccharide and Lycium barbarum polysaccharide, for which TLR4-dependent signalling (recruitment of MyD88 and activation of NF-κB and MAPK, with transcription of IL-1β, IL-6 and TNF-α) has been reported (114, 115, 118–120). Such evidence demonstrates dependence of the cellular response on TLR4; it does not by itself establish direct receptor–ligand binding, and the wording has been limited accordingly (‘TLR4-dependent signalling’ rather than receptor-complex interaction), because the cited primary studies document downstream signalling or blocking rather than receptor-level binding. This assignment carries an unavoidable interpretative burden. Plant polysaccharides extracted from non-sterile raw material routinely contain bacterial endotoxin at concentrations sufficient to activate TLR4 in isolation, and endotoxin is not removed by ethanol precipitation or by conventional dialysis (116). A TLR4-dependent effect can therefore be attributed to the polysaccharide itself only where endotoxin has been quantified below a stated limit (for example <0.1 EU/mg), or where activity persists after polymyxin B treatment, or where the response is restored when TLR4 is re-expressed in *Tlr4*-deficient cells — the last demonstrating receptor dependence rather than direct binding, and not excluding residual LPS acting through the same receptor. A minority of published studies satisfy these conditions, and this single methodological gap qualifies a substantial fraction of the TLR4 literature in this field.

Where such controls have been applied, some evidence remains for genuine engagement, though the mechanisms are probably indirect. Cooperativity with CD14 and MD-2, or perturbation of membrane lipid-raft organisation, has been proposed (121); however, the cited study addresses LPS-induced TLR4–raft signalling generally, and it should not be read as an established mechanism of action for any specific TCMP. Certain preparations also recruit the TRIF adaptor, leading to IRF3/IRF7 phosphorylation and type I interferon induction, a response of obvious relevance to antiviral vaccination since type I interferons prime cytotoxic T lymphocyte responses and establish an antiviral state in target tissue (122). Application of this property to FMDV and avian influenza vaccines has been reported (123), although in the avian case the sensing repertoire differs from the mammalian one — chickens possess TLR15 and TLR21 with no mammalian orthologue, and their accessory-receptor configuration differs from the mammalian one (99) — so the pathway cannot be assumed to be conserved.

#### 3.1.2 C-type lectin receptors

C-type lectin receptors recognise carbohydrate structure more specifically than TLRs, and Dectin-1 is the canonical sensor of β-(1→3)/(1→6)-glucans, such as those of *Ganoderma lucidum* and carboxymethylated pachyman from *Poria cocos* (124, 125). Ligand binding recruits spleen tyrosine kinase (Syk) to the ITAM-like cytoplasmic motif, assembling the CARD9–Bcl10–MALT1 signalosome and driving NF-κB-dependent transcription of IL-1β, IL-6, and IL-23 (126); this cytokine combination favours Th17 differentiation (127), which is functionally aligned with mucosal defence and with protection against extracellular bacteria and fungi. Signalling through this axis is, however, conditional on ligand geometry rather than composition alone: Dectin-1 clustering requires a rigid multivalent ligand, so triple-helical glucans are active while their single-chain counterparts of identical primary structure are markedly less so (110). The frequent omission of solvent history and aggregation state from published methods is thus not a trivial reporting deficiency but a loss of the variable that determines the outcome.

The mannose receptor (CD206) and DC-SIGN (CD209) recognise mannosylated and fucosylated epitopes present in many plant and fungal preparations, and contribute principally to internalisation and processing, supporting presentation by both MHC class I and class II pathways (128). Concurrent engagement of Dectin-1 and TLR2 or TLR4 by structurally complex material amplifies cytokine output and dendritic-cell maturation beyond the sum of the separate stimuli (129). This collaborative signalling is a plausible explanation for the potency of crude fractions relative to purified ones, but it is equally consistent with contaminant-driven synergy, and the two possibilities have rarely been distinguished experimentally.

#### 3.1.3 The NLRP3 inflammasome

The cytosolic NLRP3 inflammasome constitutes a third recognition route, engaged not by chemical epitope but by physical form. Particulate or poorly degradable material taken up by antigen-presenting cells can permeabilise the lysosomal membrane, releasing cathepsin B into the cytosol (130); the resulting danger signal nucleates the NLRP3–ASC–pro-caspase-1 complex, and active caspase-1 cleaves pro-IL-1β and pro-IL-18 to their mature forms (131). IL-1β recruits neutrophils and monocytes to the injection site, while IL-18 supports IFN-γ synthesis and cellular immunity (132). It is nevertheless essential to distinguish three claims that are often conflated: *in vitro* inflammasome activation; *in vitro* antiviral activity; and vaccine-mediated protection in a target species. For example, Isatis root polysaccharides inhibit PRRSV replication *in vitro* through TLR3/TLR4-related effects (133), which is not evidence of NLRP3-dependent vaccine protection. A claim that TCMPs enhance antimycobacterial protection in cattle is likewise not supported by studies of mycobacterial glycoconjugates themselves (134) and has been removed.

Two points temper this account. First, potassium efflux and mitochondrial reactive oxygen species are as well supported as the cathepsin B route for particulate activators generally, and the lysosomal-rupture model has been assumed rather than demonstrated for most TCMPs (135). Second, the inflammasome axis links potency to inflammation: material that activates NLRP3 efficiently is also the material most likely to produce injection-site reaction, and in food-producing animals a persistent granuloma at the injection site carries a direct carcass-quality penalty. The inflammasome is thus best understood as the node at which the physicochemical state of a preparation is converted into both its adjuvant potency and its principal liability, and this trade-off — not the pathway itself — is what requires optimisation.

### 3.2 Maturation of antigen-presenting cells and bridging to adaptive immunity

#### 3.2.1 Recruitment and uptake

Receptor engagement at the inoculation site is followed by chemokine-driven recruitment of monocytes, neutrophils, and immature dendritic cells. TCMPs increase local CCL2, CCL3, and CXCL8 expression, and their particulate fractions are internalised by phagocytosis, while soluble fractions enter by receptor-mediated endocytosis; the two routes differ in the intracellular compartment reached, and hence in the presentation pathway favoured (136). For oral delivery, recruitment and uptake take place instead at the intestinal mucosa, where the particulate and soluble fractions are sampled by microfold (M) cells of the follicle-associated epithelium and by dendritic cells of the gut-associated lymphoid tissue (Section 3.3).

#### 3.2.2 Phenotypic maturation

Maturation is conventionally documented as up-regulated MHC-II together with CD80, CD86, and CD40, reduced endocytic capacity, and increased migration towards CCL19 and CCL21. These co-stimulatory molecules provide the second signal that, with antigen recognition, permits full activation of naïve T cells and averts anergy (137). It should be noted that surface-marker up-regulation is a necessary but not sufficient criterion; functional maturation is established only by demonstrating allogeneic or antigen-specific T-cell priming, an endpoint reported in a minority of TCMP studies.

#### 3.2.3 Antigen processing and cross-presentation

Certain TCMPs modulate intracellular antigen trafficking such that exogenous antigen escapes the phagosome, is processed by the proteasome, and is loaded onto MHC class I for presentation to CD8^+^ T cells (138). Cross-presentation is the mechanistically decisive property for veterinary vaccines against intracellular pathogens such as PRRSV and PCV2, where antibody alone is insufficient for clearance (62, 139). Direct evidence in porcine dendritic cells nevertheless remains scarce, and most cross-presentation data derive from murine bone-marrow-derived dendritic cells with model antigens.

#### 3.2.4 Cytokine signature and polarisation of the adaptive response

The combination of receptors engaged determines the cytokine signature of the responding dendritic cell and thereby the direction of helper T-cell differentiation (**Figure 3**). TLR4-activating preparations typically induce IL-12 and a Th1-biased response appropriate to intracellular and viral challenge, whereas Dectin-1 agonists promote IL-23 and IL-1β and favour Th17 polarisation suited to mucosal and extracellular defence (140, 141). The practical value of this correspondence lies in its predictive use: since linkage type largely determines receptor usage (Section 2.5), the desired polarisation can in principle be specified at the stage of raw-material and fraction selection rather than discovered empirically. Two constraints limit how far this can be taken at present. The outcome of Dectin-1 engagement depends on the physical form of the ligand (particulate presentation and particle size alter the response, and soluble glucans can bind Dectin-1 without necessarily triggering signalling (142) so receptor activation cannot be inferred from chain length or triple-helical structure alone, and a general dose–response rule for Dectin-1 agonists cannot yet be stated. Second, the IL-12/IL-23 axis is not equivalently configured across species, ruminant and avian responses to identical stimuli differing in both magnitude and Th subset dominance (143).

**Figure 3:**
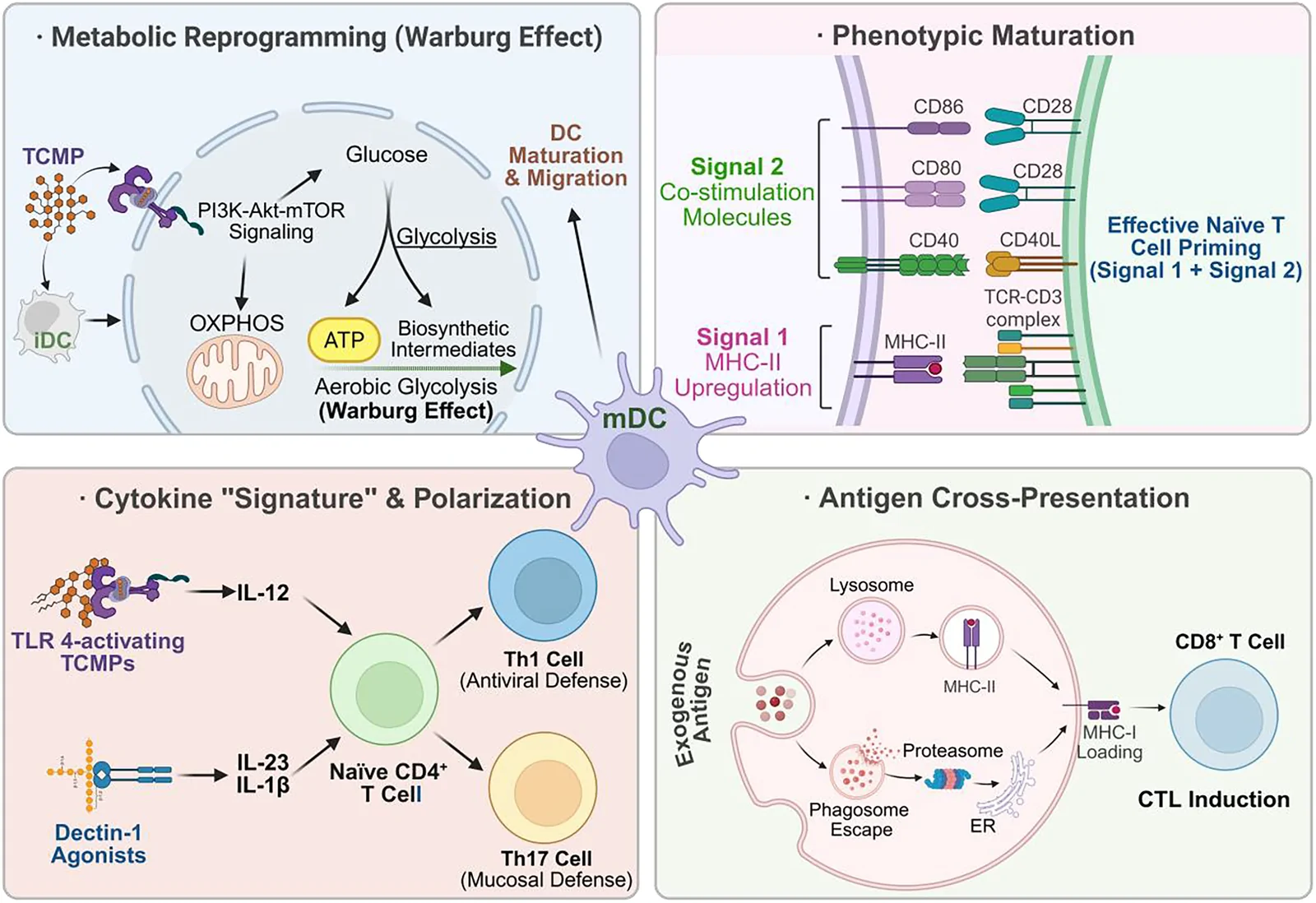
Orchestrated programming of dendritic cells (DCs) by TCMPs as vaccine adjuvants. This illustration depicts the orchestrated programming of DCs by TCMPs as vaccine adjuvants, encompassing four interconnected dimensions. First, TCMPs induce metabolic reprogramming, shifting DCs from mitochondrial oxidative phosphorylation to aerobic glycolysis, thereby enhancing activation and migration. Second, TCMPs promote phenotypic maturation of DCs, characterized by robust upregulation of co-stimulatory molecules (CD80, CD86, CD40) and MHC-II molecules, ensuring effective T cell priming. Third, certain TCMPs enhance antigen processing and cross-presentation, facilitating the induction of cytotoxic T lymphocytes (CTLs), which is crucial for veterinary vaccines targeting intracellular pathogens. Lastly, the specific combination of pattern recognition receptors (PRRs) engaged by polysaccharides dictates the “cytokine signature” secreted by DCs, guiding the differentiation of T helper (Th) cells toward antiviral (Th1) or mucosal/extracellular (Th17) responses. Evidence code: solid arrows denote direct experimental support; dashed arrows denote inferred steps. MHC class II presentation of exogenous antigen and MHC class I cross-presentation are depicted separately; metabolic reprogramming is shown as a proposed rather than established effect of TCMPs.

#### 3.2.5 Depot behaviour, lymphatic drainage, and antigen kinetics

An aspect absent from most mechanistic treatments of TCMPs, yet central to the performance of every licensed adjuvant, is the physical fate of the antigen–adjuvant complex. High-molecular-mass acidic polysaccharides increase formulation viscosity and adsorb protein antigen through electrostatic and hydrogen-bonding interactions, slowing antigen efflux from the injection site and extending the period of antigen availability to arriving antigen-presenting cells (144). Conversely, self-assembled nanoparticulate forms in the approximate range of 20–200 nm drain efficiently to the regional lymph node and are taken up directly by resident subcapsular sinus macrophages and dendritic cells, favouring rapid germinal-centre engagement (145). The two behaviours are not merely different but partly opposed, and the choice between them should follow the immunological requirement — sustained antibody affinity matuation, or rapid onset of cell-mediated immunity.

This dimension also explains a recurrent and otherwise puzzling observation: liposomal or nanoparticulate encapsulation improves the performance of *Lycium* and *Ganoderma* polysaccharides far more than would be predicted from any change in receptor affinity (88–90, 106). The gain is largely pharmacokinetic, and its corollary is methodological — without an empty-carrier control of matched composition, such experiments quantify the formulation rather than the polysaccharide.

### 3.3 Mucosal immunity and the gut–lung axis

Oral administration is the route of greatest practical interest in livestock production, since it avoids handling stress and individual injection, and it engages a distinct set of mechanisms.

#### 3.3.1 Induction of secretory IgA

Structurally stable polysaccharides, including those of *Dioscorea opposita* and *Ganoderma lucidum*, resist gastric acid and mammalian glycosidases and reach the small intestine substantially intact (146, 147). Transcytosis by microfold cells delivers them to Peyer’s patches, where proliferation and class-switching of IgA^+^ B cells is induced, and secretory IgA released by lamina propria plasma cells neutralises pathogens before epithelial penetration (**Figure 4A**) (148). Acidic pectic fractions are particularly effective in this compartment, consistent with their adhesion to the mucus layer through uronic acid residues.

**Figure 4:**
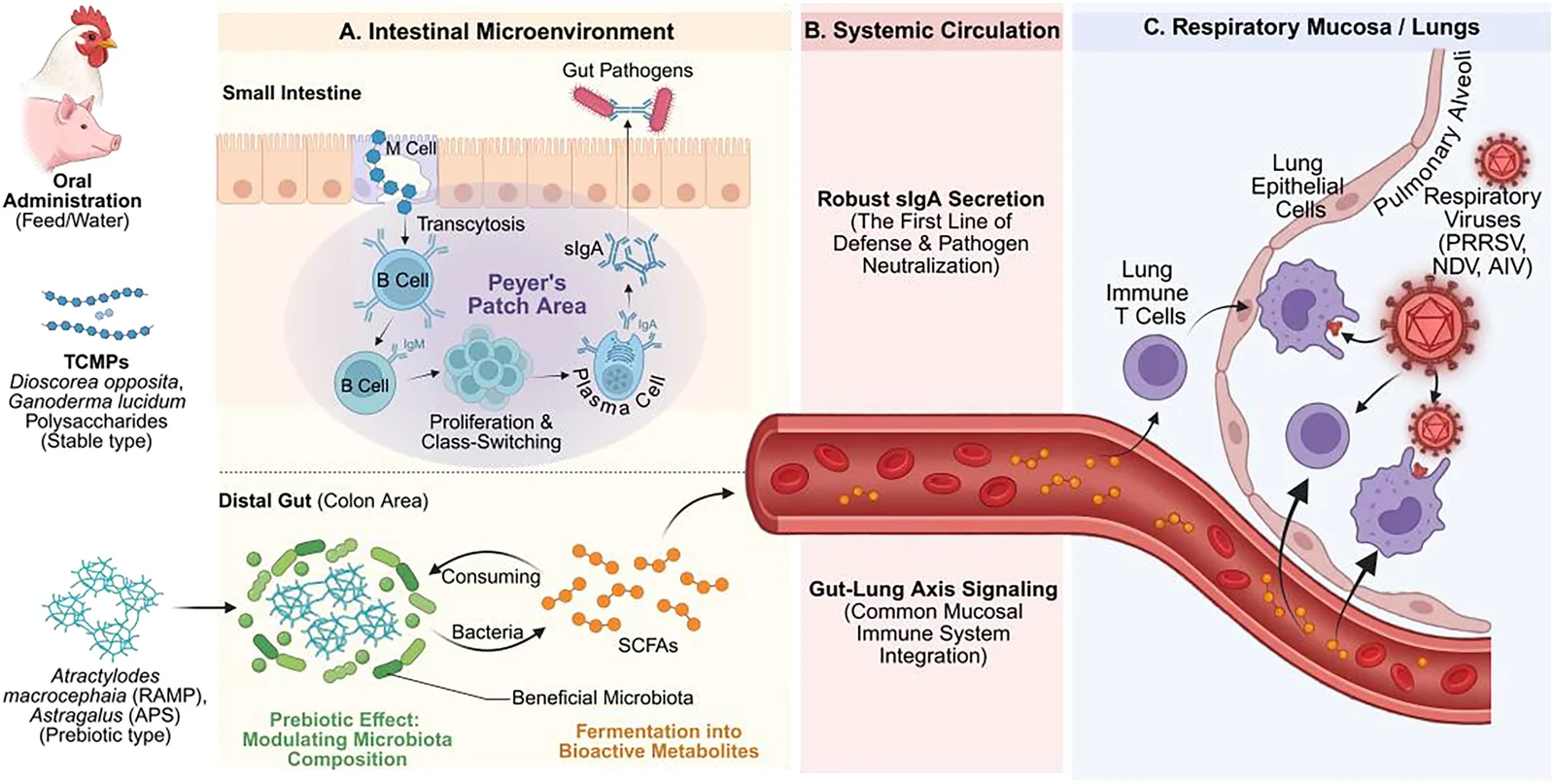
Schematic diagram illustrating the mechanisms underlying oral TCMP-mediated regulation of the gut-lung axis mucosal immunity. **(A)** Intestinal microenvironment: oral administration of TCMPs leads to dual effects in the gut. In the Peyer’s patches of the small intestine (upper panel), stable TCMPs (e.g., from Ganoderma lucidum) are actively transcytosed by microfold cells (M cells). They stimulate IgA^+^ B cells to differentiate into plasma cells, promoting robust secretion of secretory IgA (sIgA). sIgA serves as a frontline defense by neutralizing gut pathogens in the mucus layer. In the distal intestine (lower panel), prebiotic-like TCMPs (e.g.), RAMP from Atractylodes macrocephala, APS from Astragalus) modulate the microbiota composition by promoting beneficial bacteria (e.g., Lactobacillus, Bifidobacterium). Fermentation of these polysaccharides yields bioactive metabolites, specifically short-chain fatty acids (SCFAs). **(B)** Systemic circulation: SCFAs produced in the gut cross the intestinal epithelium, enter the systemic vasculature, and travel to distant mucosal sites, representing the integration of the common mucosal immune system. **(C)** Respiratory mucosa & lungs: gut-derived SCFAs reach the alveolar structures in the lungs via circulation. They prime respiratory immune cells, such as alveolar macrophages and T cells, enhancing their responsiveness. The primed immune cells exhibit enhanced protective capacity against respiratory viral infections (e.g., NDV, AIV, PRRSV), thereby completing the functional circuitry of the gut-lung axis. Created by BioRender. The figure is a cross-species conceptual model, not a single-host experiment; the microbiota→SCFA→circulatory→respiratory-priming sequence is drawn with dashed lines because the causal chain has not been closed experimentally, and direct actions of polysaccharides on gut-associated lymphoid tissue provide an alternative explanation.

#### 3.3.2 Microbial fermentation and distal mucosal priming

Indigestible fractions such as *Atractylodes* RAMP and *Astragalus* APS act as fermentable substrates in the distal intestine, shifting community composition towards *Lactobacillus* and *Bifidobacterium* (149, 150). Short-chain fatty acids generated by fermentation enter the circulation and act on immune cells at anatomically remote mucosal surfaces, including the respiratory tract (**Figure 4B**) (151). Within the framework of the common mucosal immune system, this provides a coherent explanation for improved protection against respiratory pathogens such as Newcastle disease virus and avian influenza virus following oral administration (**Figure 4C**).

The mechanism is plausible and the outcome reproducible, yet the causal chain is rarely closed. Demonstrating that a systemic effect is microbiota-dependent requires germ-free, antibiotic-depleted or faecal-transfer controls, or at minimum measurement of the implicated metabolites in portal or systemic blood; none of these is standard in the veterinary literature, and direct engagement of gut-associated lymphoid tissue by intact polysaccharide remains an equally admissible explanation. The two mechanisms have different practical implications — dose and timing requirements differ substantially between a receptor-mediated and a fermentation-mediated effect — so the distinction is not academic. It is also worth noting that fermentation-based effects are inherently sensitive to the composition of the basal diet and to antibiotic use, which may account for part of the between-farm variability reported for oral polysaccharide supplementation.

### 3.4 Species specificity and the evidential status of mechanistic claims

The mechanisms described above are drawn overwhelmingly from murine models, and their transfer to livestock is neither automatic nor uniformly justified. Several differences are structural rather than quantitative. The chicken genome lacks a *CLEC7A* (Dectin-1) orthologue, so avian β-glucan recognition must proceed through alternative lectins or TLRs (99). Ruminants and pigs possess large circulating populations of WC1^+^ γδ T cells with scavenger-receptor-like WC1 co-receptors that have no murine equivalent and that respond directly to certain carbohydrate structures (152). Porcine alveolar macrophages are unusually abundant and are the principal target cell of PRRSV, which places them at the centre of any adjuvant strategy in that species, while its murine equivalent lacks the PRRSV host-susceptibility and virus–macrophage interaction context (153). Bovine and porcine dendritic-cell subsets differ from murine subsets in both surface phenotype and cytokine output, and the murine convention of classifying responses as strictly Th1 or Th2 maps poorly onto ruminant immunity (143).

The consequence is that mechanistic and efficacy evidence should be recorded separately by species and by route, rather than aggregated. **Table 1** presents the principal preparations discussed in Section 2 in this stratified form, distinguishing the species in which an adjuvant effect has been demonstrated from the species in which the proposed mechanism has actually been interrogated, and assigning each an evidence tier (**Box 1**). Two patterns emerge. First, for the majority of preparations, the mechanistic account derives from mice or immortalised murine cell lines, while the efficacy data derive from poultry or pigs; the mechanism is therefore inferential for the target species in most cases. Second, even the preparations for which target-species data are most complete — for example, *C. militaris* polysaccharides in Newcastle disease vaccination of chickens, and β-glucans examined mechanistically in porcine cells — rest on structurally defined material, which again suggests that the principal obstacle is chemical definition rather than experimental access to livestock.

**Table 1:** Study-level evidence summary for the principal preparations discussed in this review, stratified by evidence tier (**Box 1**).

Preparation	Key structural features	Tier	Species of efficacy (antigen)	Species of mechanism	Refs
Astragalus polysaccharide (APS)	α-1, 4/1, 6-glucan + acidic arabinogalactan	T2; T1 (fish)	Chicken (NDV, AIV); pig (FMDV); fish (Edwardsiella, Vibrio)	Mouse; chicken (partial); pig (inferential)	(36–47)
Ginseng polysaccharide (GPS)	Starch-like glucan + pectic acidic fraction	T2	Chicken (AIV, NDV); pig (FMDV, PRRSV)	Mouse	(44, 52–55)
Codonopsis pilosula polysaccharide (CPP)	Pectic (GalA, Ara)	T4	— (feed-supplement study in pigs)	Bovine macrophage (*in vitro*)	(59, 60)
Atractylodes macrocephala polysaccharide (RAMP/PAMK-NLC)	Heteroglycan (Rha, Ara, Xyl, Man, Glc, Gal)	T2 (poultry); T3 (mouse)	Chicken (ND); mouse (FMDV, OVA)	Mouse	(65–69)
Polygonatum sibiricum polysaccharide (PSP)	β-fructan/β-galactan (processing-dependent)	T4	—	*In vitro*	(70, 71)
Epimedium polysaccharide (EPS)	Heterogeneous; gut-axis modulator	T4	Piglet (with APS; sulfated derivative)	*In vitro*/mouse	(74, 75, 77)
Artemisia annua polysaccharide (AAPs)	Acidic pectin (GalA 8%—28%)	T4	Lamb (dietary whole-extract study)	*In vitro* (macrophage)	(78–80)
Lycium barbarum polysaccharide (LBP)	Arabinogalactan-protein + RG-I; peptide moiety	T3	Mouse (Chlamydophila DNA, OVA, PCV2 antigen)	Mouse DC	(90–95)
Schisandra chinensis polysaccharide (SCP)	Acidic (GalA, Ara)	T4	—	*In vitro* (TLR4)	(96, 97)
Glycyrrhiza polysaccharide	—	T2	Chicken (NDV)	—	(98)
Cuscuta chinensis polysaccharide	Acidic	T4	—	*In vitro*/mouse	(99, 100)
Ganoderma lucidum/sinense (GLP/GSP)	Branched β-glucan + glycoprotein heteropolysaccharide	T2 (chicken); T3 (mouse)	Chicken (ND); mouse (OVA, liposomal)	Mouse DC	(105–110)
Lentinan	β-(1→3)-glucan, β-(1→6) branched (triple helix)	T4	— (licensed human therapeutic)	Pig cells (*in vitro*, IFN-γ)	(111, 112)
Cordyceps militaris polysaccharide	Mannoglucan/galactomannan	T2	Chicken (NDV)	—	(115, 116)
Poria cocos (pachyman)	β-(1→3)-glucan (derivatised)	T4	—	*In vitro*	(117)
Isatis indigotica (leaf)	Degraded polysaccharide	T4	—	*In vitro* (PRRSV)	(81, 139)

Each row is traceable to the representative primary references listed. Tier denotes the highest level of vaccine-relevant evidence identified; — indicates that no vaccine-context study was identified.

Nothing in this assessment implies that murine work is dispensable; it remains the only practical setting for receptor knockout and adoptive-transfer approaches. What it does imply is that a mechanism established in mice should be reported as a hypothesis for the target species until tested in homologous cells, and that the minimum confirmatory experiment — a receptor-blocking or knockdown study in porcine, bovine, or avian primary antigen-presenting cells — is well within the reach of most veterinary immunology laboratories.

## 4 Species-specific applications in livestock and poultry

Pattern recognition receptor repertoires, lymphoid tissue organisation and dominant lymphocyte subsets differ appreciably among domestic species, having been shaped by divergent evolutionary pressures and physiological environments. An adjuvant that performs well in one production species therefore cannot be assumed to perform equivalently in another, and the more useful question is not which polysaccharide is most potent but which immunological deficit needs correcting in a given host–pathogen system. The four settings below differ precisely in that respect: virus-induced macrophage immunosuppression in pigs, an unusually large innate-like T-cell compartment in ruminants, a mucosally dominated and Dectin-1-deficient immune system in poultry, and temperature-dependent, largely innate immunity in farmed fish.

### 4.1 Swine: targeting porcine alveolar macrophages to counter immunosuppression

Porcine alveolar macrophages (PAMs) are the principal target of PRRSV, which establishes immunosuppression by shifting macrophage polarisation towards an M2-like phenotype with elevated IL-10 output, blunting both innate antiviral function and subsequent T-cell priming (154). This makes PAM reprogramming, rather than antibody enhancement alone, the rational objective of adjuvant design in pigs. β-Glucans engage the Dectin-1/Syk axis in mononuclear phagocytes and enhance T-cell IFN-γ responses in cells derived from PRRSV-infected pigs (108). Whether this translates into a reproducible reversal of PRRSV-induced M2 polarisation *in vivo* is not established; PAM reprogramming therefore remains a plausible but as yet unproven objective.

The porcine setting is also the one in which the limits of the current evidence are most clearly exposed. PRRSV vaccine efficacy is conventionally judged by viraemia and lung lesion scores after homologous challenge, yet the field problem is heterologous protection, and no polysaccharide adjuvant has been evaluated against a genetically distant challenge strain. Immunological improvement measured as cytokine ratio or antibody titre has repeatedly failed to predict cross-protection for this virus (155), so favourable *in vitro* repolarisation data should not be presented as evidence of field utility. A second consideration is specific to the species: the greater part of commercial pig vaccination occurs in the first three weeks of life, when maternally derived antibody interferes with priming, and whether polysaccharide adjuvants mitigate or exacerbate that interference has not been examined. Given that stronger innate activation may accelerate antigen clearance in the presence of maternal antibody, the question cannot be settled by analogy.

### 4.2 Ruminants: non-conventional activation of γδ T cells

Cattle and sheep possess γδ T cell frequencies of up to approximately 40% of circulating T cells in young animals, against 1–5% in humans and mice (156). These cells express WC1, a scavenger-receptor cysteine-rich molecule with no rodent orthologue, and respond to certain carbohydrate and phosphoantigen structures without MHC restriction, secreting IFN-γ rapidly and exerting direct cytotoxicity (152). Adjuvant materials capable of enhancing bovine γδ T-cell responses have been reviewed (157), and WC1 has been characterised as a co-receptor/pattern-recognition receptor on bovine γδ T cells (158). However, neither source provides direct primary evidence that a specific plant polysaccharide (for example from *Euterpe oleracea* or *Atractylodes macrocephala*) activates bovine γδ T cells through a defined WC1–TLR2 pathway; the material, cell type and receptor mechanism must each be supported separately, and this is therefore treated here as a hypothesis rather than an established mechanism. The immunological logic is attractive: an adjuvant capable of engaging a numerically dominant, rapidly responding, MHC-unrestricted compartment could provide early containment while conventional adaptive responses develop, which is of particular value in neonatal calves whose αβ T-cell responses are immature.

The supporting evidence is nevertheless thinner than the mechanistic argument implies. Most WC1-related work has been performed with peripheral blood mononuclear cells *in vitro*; demonstration that γδ activation by a polysaccharide translates into improved vaccine-induced protection *in vivo* is, to date, absent. Ruminant vaccinology also imposes constraints that receive little attention in this literature. Bovine respiratory disease and FMDV vaccines are administered to animals whose rumen degrades orally delivered polysaccharide before it reaches the intestine, which largely nullifies the oral route that makes these materials attractive in monogastrics — a limitation not acknowledged in the original framing of the gut–immune axis. Injectable use is thus the realistic option in adult ruminants, whereas oral administration remains plausible only in pre-ruminant calves and lambs, in which the reticular groove reflex permits bypass of the rumen (159).

### 4.3 Poultry: mucosal delivery and the constraints of a distinct innate repertoire

Poultry production offers the most favourable conditions for polysaccharide adjuvants and, simultaneously, the least transferable immunological background. On the practical side, birds are vaccinated in very large cohorts by drinking water, spray, or feed, so an orally active adjuvant requiring no individual handling has immediate commercial value; the majority of published *in vivo* efficacy data for TCMPs accordingly derive from chickens, with *Astragalus* polysaccharide, *Ganoderma* polysaccharide, and *Atractylodes* RAMP all shown to raise Newcastle disease and avian influenza antibody titres and to increase intestinal secretory IgA (36, 37, 43, 44). In ovo injection of Astragalus polysaccharide accelerated the onset of Newcastle disease immunity (38), an outcome of real value given the narrow window between hatch and field exposure.

Gut-associated lymphoid tissue in birds is organised differently from that of mammals: caecal tonsils, Peyer’s patches, Meckel’s diverticulum, and the bursa of Fabricius together perform functions distributed otherwise in mammals, and the bursa provides a site of B-cell development with no mammalian counterpart (160). More consequentially for mechanistic interpretation, the chicken genome lacks a clear CLEC7A orthologue, so the Dectin-1/Syk pathway invoked for β-glucan activity in pigs and mice cannot be assumed to operate in the same form (99). The alternative pathway is not established: TLR15 is a protease-sensing receptor and TLR21 a CpG-DNA receptor, and neither should be listed as a β-glucan receptor without direct evidence from chicken-derived cells; TLR2 remains only a candidate (99). That β-glucans nonetheless enhance avian vaccine responses is well documented (109); the pathway responsible is not established, and its assignment by extrapolation should be avoided. Interpretation is complicated further by the fact that many avian trials combine vaccination with dietary supplementation, so improved titres may reflect better nutritional and intestinal status rather than adjuvant action in the strict sense — a distinction that requires a pair-fed or isocaloric control arm.

### 4.4 Aquaculture

Farmed fish represent the setting in which polysaccharide immunostimulants are already in routine commercial use, yet they are almost absent from reviews of veterinary adjuvants. *Astragalus* polysaccharide improved survival after *Edwardsiella ictaluri* and *Vibrio harveyi* challenge when administered with bacterial antigen (45, 46), and β-glucans are incorporated into commercial feeds for salmonids and carp (161). Teleost immunity is heavily innate-weighted, with limited affinity maturation and slow, temperature-dependent antibody responses, so an adjuvant acting principally on macrophage and granulocyte function is comparatively well matched to the host (162). Oral and immersion delivery are also operationally straightforward at production scale.

Two qualifications apply. Protection in fish is frequently reported as relative percentage survival after challenge without accompanying immunological measurements, which makes it difficult to distinguish genuine adjuvant activity from non-specific trained-innate stimulation of a kind that would occur without antigen; and prolonged β-glucan feeding has been associated in some studies with immune exhaustion and reduced growth, indicating that the pulsed regimens used commercially reflect empirical necessity rather than optimisation (163). Aquaculture nevertheless provides the largest body of field-scale experience with these materials, and its dosing practice deserves closer examination by those working in terrestrial species.

### 4.5 The gap between experimental demonstration and field application

Across all four settings, a consistent asymmetry is apparent between the immunological sophistication of the mechanistic work and the design of the efficacy studies. Four deficiencies recur and together account for the limited translation observed to date.

Comparators are usually inadequate. The majority of trials compare an adjuvanted vaccine with unadjuvanted antigen or with saline, rather than with the aluminium salt or oil-in-water emulsion actually used in the field; superiority over no adjuvant is not a meaningful commercial claim, and the few head-to-head comparisons available suggest that polysaccharides are best positioned as components of combination formulations rather than as replacements for oil emulsions in inactivated viral vaccines (164).

Endpoints are predominantly immunological rather than protective. Antibody titre and cytokine expression are convenient but imperfect correlates, and for several of the pathogens concerned, the relationship between titre and protection is weak. Challenge studies, or field trials with morbidity, mortality, and production endpoints, remain uncommon.

Study duration is short relative to production cycles. Most reports terminate within four to six weeks of vaccination, whereas breeding stock require protection over months, and maternal antibody transfer to progeny is a principal objective in poultry and pig breeder vaccination. Durability of polysaccharide-adjuvanted responses is therefore largely undocumented.

Finally, dose selection is rarely justified. Doses are commonly chosen from a single earlier study or from murine work, despite the non-monotonic dose–response behaviour characteristic of these materials (Section 2.5) and the possibility of regulatory rather than effector outcomes at high dose. Reports of ineffectiveness may in some instances reflect overdosing rather than the absence of activity, a possibility that cannot be assessed without full dose–response data.

None of these problems is intrinsic to polysaccharide adjuvants; each is a feature of how they have been evaluated. Addressing them requires no new chemistry, only the trial design conventions already standard in veterinary vaccine registration, and the priorities identified here inform the research agenda proposed in Section 5.

## 5 Challenges and future prospects in veterinary medicine

The global movement to reduce and replace antibiotics in animal production has created a policy environment favourable to immunomodulatory feed and vaccine components, and Chinese medicine polysaccharides are frequently presented as beneficiaries of that shift. Opportunity alone, however, does not constitute a development pathway. Four decades of publication have produced a large and internally consistent body of evidence that these materials modulate immune responses, yet, to our knowledge, no TCMP-based adjuvant has been licensed as a component of a registered veterinary vaccine in any major jurisdiction as of the time of writing. This statement concerns TCMP adjuvants specifically: other polysaccharide-adjuvant chemistries have progressed further, most notably delta-inulin (Advax™), a plant-derived polysaccharide adjuvant that has advanced through veterinary and clinical development (165). That contrast is instructive (it is the defined, consistent, synthetically controllable material that has advanced) and it frames the central problem for this field: not biological activity, but chemical definition. It is also essential here to distinguish approval as a drug from approval as a vaccine adjuvant; pharmacopoeial listing of a polysaccharide injection as a therapeutic does not constitute adjuvant licensure. That discrepancy is the central problem for the field, and it is more instructive to ask why translation has failed than to reiterate the potential.

### 5.1 Chemical definition and batch consistency

The primary obstacle is regulatory rather than immunological. Vaccine adjuvants are assessed as defined chemical entities with specified identity, purity, and stability, and a hot-water extract of a botanical raw material cannot meet that standard. Polysaccharide composition varies with cultivar, geographical origin, harvest season, drying method, and extraction conditions, and the resulting variation in molecular mass distribution and uronic acid content is sufficient to alter potency (Section 2.5). Consistency of the kind demanded by a marketing authorisation therefore requires either cultivation and processing under controlled conditions with release specifications tied to structural parameters, or a shift to defined single-component preparations.

The second option is the more realistic and is already available in prototype. Lentinan is a chemically defined β-glucan produced under pharmaceutical conditions, and enzymatic or chemo-enzymatic synthesis now permits the preparation of β-(1→3)-glucans with controlled chain length and branching (166). Structurally defined, reproducibly manufactured oligo- and polysaccharides eliminate the batch-variability objection entirely, at the cost of abandoning the framing of these materials as natural extracts. Whether a field organised around botanical provenance is prepared to make that transition is a question about research culture rather than about science, but the regulatory arithmetic strongly favours definition over authenticity.

### 5.2 Safety assessment appropriate to food-producing animals

Long dietary use of the source material is routinely offered as evidence of adjuvant safety. The inference does not hold, for three reasons that are specific to this application. Parenteral administration bypasses the gastrointestinal barrier that conditions the safety record of oral consumption; chemical derivatisation, which frequently confers potency, creates entities without any dietary history (Section 2.5); and residue and withdrawal considerations differ from those governing food additives. Injection-site reactions require particular attention, since granulomatous change at the site of vaccination carries a direct carcass-value penalty and is mechanistically linked to the same inflammasome activation that contributes to potency (Section 3.1.3). Repeated-dose toxicity, local tolerance, and residue depletion data generated in the target species are prerequisites for registration, and are almost entirely absent from the current literature. Consequently, and in contrast with the frequent assertion of high biosafety, the safety profile of these materials as parenteral veterinary adjuvants should be regarded as insufficiently characterised rather than as established.

### 5.3 Trial design and evidentiary standards

The deficiencies identified in Section 4.5 — inadequate comparators, immunological rather than protective endpoints, short observation periods, and unjustified dose selection — are correctable without new chemistry. A minimal design capable of supporting a development decision would include a licensed adjuvant comparator, a full dose–response arm sufficient to detect non-monotonic behaviour, challenge or field protection as the primary endpoint, an observation period matched to the production cycle of the species concerned, and endotoxin quantification of the test article. For orally administered preparations, a pair-fed control is additionally necessary to separate adjuvant action from improved nutritional and intestinal status. Adoption of these conventions would allow accumulated observations to be compared rather than merely counted.

### 5.4 Delivery systems and administration routes

Automated, non-invasive administration is likely to dominate future veterinary vaccination because it reduces labour cost and handling stress (167), and polysaccharides are well suited to this direction: acid-resistant, enzyme-stable core–shell constructs based on chitosan or modified starch can protect antigen during gastric transit while exploiting the mucoadhesive properties of the carrier to prolong intestinal residence. Film-forming and permeation-enhancing fractions may also support transdermal patches and spray delivery.

Realism about the pharmacokinetic contribution is nevertheless required. Much of the improvement attributed to encapsulated polysaccharides reflects altered biodistribution rather than receptor engagement (Section 3.2.5), and where a carrier is itself immunologically active, the contribution of the polysaccharide cannot be bounded without an empty-carrier control of matched composition. Species-specific physiology imposes further limits: the rumen degrades orally delivered polysaccharide in adult cattle and sheep, restricting the oral route to pre-ruminant animals (Section 4.2), and gastric pH, transit time and intestinal glycosidase profiles differ sufficiently among pigs, poultry and fish that a formulation optimised in one species requires re-optimisation in another.

### 5.5 Compatibility with disease surveillance and eradication programmes

The capacity to differentiate infected from vaccinated animals underpins the eradication of major epidemic diseases, and any new adjuvant must be shown not to interfere with it. Verification that polysaccharide-adjuvanted responses do not generate antibodies cross-reactive with diagnostic antigens is a straightforward but non-optional requirement, and is particularly relevant where structurally complex plant material carries glycan epitopes shared with pathogen surfaces. Proposals for traceable adjuvants bearing covalently attached fluorescent or isotopic labels would permit *in vivo* tracking of distribution and clearance; such conjugates are, however, new chemical entities requiring their own safety and residue assessment, and their utility is likely to be confined to development studies rather than commercial product.

### 5.6 Cost, raw material supply, and process sustainability

Cost tolerance in veterinary biologics is low, and margins in commodity meat production leave little room for premium adjuvants. Two routes to viable cost of goods are apparent. The first is the use of agro-processing by-products — corncobs, fruit pomace, seed cake, and spent herbal residue — which are available at a small fraction of the price of cultivated medicinal root and which, in the case of fruit and seed residues, retain pectic fractions of demonstrated activity (Section 2.3.3). Leaf material offers a related advantage, being harvestable without destroying the plant and therefore renewable on an annual cycle (Section 2.2.3). The second is process substitution: replacing energy-intensive hot-water extraction and organic-solvent precipitation with enzyme-assisted extraction, ultrasound- or microwave-assisted methods, or deep eutectic solvents reduces both cost and environmental burden.

Neither route is without complication. By-product streams are more variable in composition than cultivated raw material, which aggravates precisely the consistency problem described in Section 5.1; and residual solvent, pesticide, and mycotoxin burdens in agricultural waste must be controlled to standards appropriate for parenteral use. Cost reduction and chemical definition therefore pull in opposite directions, and the resolution most likely to succeed is process intensification applied to a defined, reproducibly sourced fraction rather than valorisation of whatever material happens to be cheap.

## 6 Conclusion

Polysaccharides derived from Chinese medicine herbs and medicinal fungi modulate innate and adaptive immune responses through defined receptor systems, and enhance vaccine-induced immunity in pigs, ruminants, poultry, and farmed fish under experimental conditions. The evidence assembled here supports three conclusions of practical consequence. Activity is determined by molecular architecture — glycosidic linkage, molecular mass, branching, charge, and conformation — rather than by botanical origin, so classification by plant part, although convenient, has no mechanistic standing and should not guide candidate selection. Mechanistic accounts remain largely inferential for the target species, since the receptor pathways invoked have been characterised chiefly in mice while efficacy has been demonstrated chiefly in poultry and pigs, and structural differences such as the absence of a Dectin-1 orthologue in chickens and the prominence of WC1^+^ γδ T cells in ruminants preclude straightforward extrapolation. Translation has been limited not by insufficient biological activity but by insufficient chemical definition and by trial designs that do not address the questions on which registration depends. Claims that these materials establish durable germinal-centre-dependent humoral memory or tissue-resident memory T cells are not supported by direct evidence and are presented here as hypotheses requiring validation.

The corresponding priorities follow directly. Structurally defined preparations, whether purified fractions or enzymatically synthesised glucans, should replace crude extracts in mechanistic and efficacy work; endotoxin quantification and receptor-dependence testing in homologous primary cells should become routine; and efficacy studies should employ licensed adjuvant comparators, full dose–response arms, and protection endpoints over production-relevant intervals. Safety data adequate for parenteral use in food-producing animals must be generated in the target species rather than inferred from dietary history. Addressed in this order, these requirements are ordinary rather than formidable, and their fulfilment would determine whether polysaccharide adjuvants become a component of veterinary vaccine formulation or remain a well-documented laboratory phenomenon.
